# The long non-coding RNA NONHSAT062994 inhibits colorectal cancer by inactivating Akt signaling

**DOI:** 10.18632/oncotarget.19827

**Published:** 2017-08-02

**Authors:** Xiao-Shun He, Ling-Chuan Guo, Ming-Zhan Du, Shan Huang, Ren-Peng Huang, Sheng-Hua Zhan, Dong-Mei Gu, Wei-Shuo Liu, Xi-Ming Wang, Hua Wu, Wen-Juan Gan

**Affiliations:** ^1^ Department of Pathology, The First Affiliated Hospital of Soochow University, Soochow University, Suzhou 215006, China; ^2^ Pathology Center and Department of Pathology, Soochow University, Suzhou 215123, China; ^3^ Department of Radiology, The First Affiliated Hospital of Soochow University, Soochow University, Suzhou 215006, China

**Keywords:** LncRNA NONHSAT062994, colorectal cancer, growth, Akt signaling

## Abstract

The aberrant expression of long noncoding RNAs (lncRNAs) is implicated in cancer development and progression. However, the clinical significance and mechanism by which NONHSAT062994 regulates colorectal cancer (CRC) is unknown. We here reported that NONHSAT062994 was significantly downregulated in human CRC tissues and cell lines. Moreover, its expression was inversely correlated with tumor size and overall survival (OS) time in CRC patients. In CRC cells, the overexpression and knockdown of NONHSAT062994 inhibited and enhanced CRC cell growth, respectively, *in vitro* and *in vivo*. Mechanistically, NONHSAT062994 functioned as a tumor suppressor to inhibit CRC cell growth by inactivating Akt signaling. Notably, the NONHSAT062994 expression status was negatively correlated with the Akt downstream targets c-Myc and Cyclin D1 in clinical CRC samples. The current findings suggest that NONHSAT062994 plays a critical role in the development of CRC by regulating Akt signaling, and identified a candidate prognostic biomarker or potential therapeutic target for CRC patients.

## INTRODUCTION

Colorectal cancer (CRC), 1 of the most common types of cancer, is associated with a high mortality rate worldwide [1]. Although great developments have been made in diagnostic and therapeutic modalities, the overall survival (OS) time of CRC patients is still unsatisfactory [2]. In most cases, CRC is diagnosed at an advanced pathological stage before symptoms appear, which may be a major cause of the poor survival. Therefore, identifying new molecular markers or factors that can be used in the clinical diagnosis of CRC patients at an early stage is critical.

Accumulating studies have implicated many molecules and signaling pathways in CRC development and progression. For example, Nur77, RARγ, and OVOL2 are abnormally expressed in CRC tissues and regulate colorectal tumorigenesis and metastasis [3–5]. Dysregulation of signaling pathways such as the Wnt/β-catenin and PI3K/Akt pathways contributes to the malignant proliferation, extensive invasion, and distant metastasis of CRC [6, 7]. Interestingly, recent studies indicated that non-coding RNAs (lncRNAs) have extensive biological roles in cellular development, differentiation, and apoptosis [8–10]. However, aberrant lncRNA expression has frequently been reported in several cancer types, including CRC [11]. For example, upregulation of the lncRNA HOTAIR in CRC enhances the invasive ability of CRC cells and contributes to poor prognosis in CRC patients [12]. In contrast, the lncRNA Loc554202 is downregulated in CRC tissues and cell lines, and functions as a tumor suppressor to regulate cell apoptosis, in part by activating caspase cleavage cascades [13].

lncRNAs exert their functions via complex and diverse molecular mechanisms including epigenetic, transcriptional, and post-transcriptional alterations. For example, the lncRNA HOTAIR regulates polycomb-dependent chromatin modification in CRC [12]. lncRNA-LALR1 inhibits *Axin1* expression by recruiting CTGF to the *Axin1* promoter region in liver cells [14]. In addition, lncRNA-ATB competitively binds to members of the miR-200 family to upregulate the expression of ZEB1 and ZEB2, which are critical regulators of the epithelial-mesenchymal transition that contributes to cancer cell invasion and metastasis [15]. Furthermore, lncRNAs can serve as signaling molecules to regulate signal transduction by directly interacting with functional domains in signaling proteins. For example, lnc-DC regulates STAT3 signaling by binding directly to STAT3 in the cytoplasm, which promotes human dendritic cell differentiation [16]. Cytoplasmic lncRNA NKILA suppresses breast cancer metastasis via the mechanism where NKILA directly interacts with NF-κB/IκB and inactivates NF-κB inflammatory signaling [17].

lncRNA NONHSAT062994 is a 585-bp transcript located on human chromosome 19. However, no studies have yet focused on its biological role and regulatory mechanism in human diseases including cancer. The aim of the current study was to investigate the role and clinical significance of NONHSAT062994 in CRC. The results demonstrated that NONHSAT062994 was significantly downregulated in both CRC tissues and cell lines. This downregulation was closely associated with a large tumor size and poor survival of CRC patients. Furthermore, *in vitro* and *in vivo* studies revealed that NONHSAT062994 inhibits CRC cell growth by inactivating Akt signaling. These findings suggest that NONHSAT062994 could be used as a new biomarker for CRC prognosis and may serve as a potential therapeutic target for CRC.

## RESULTS

### Aberrant lncRNA NONHSAT062994 expression in human CRC tissues and cell lines

**Figure 1:**
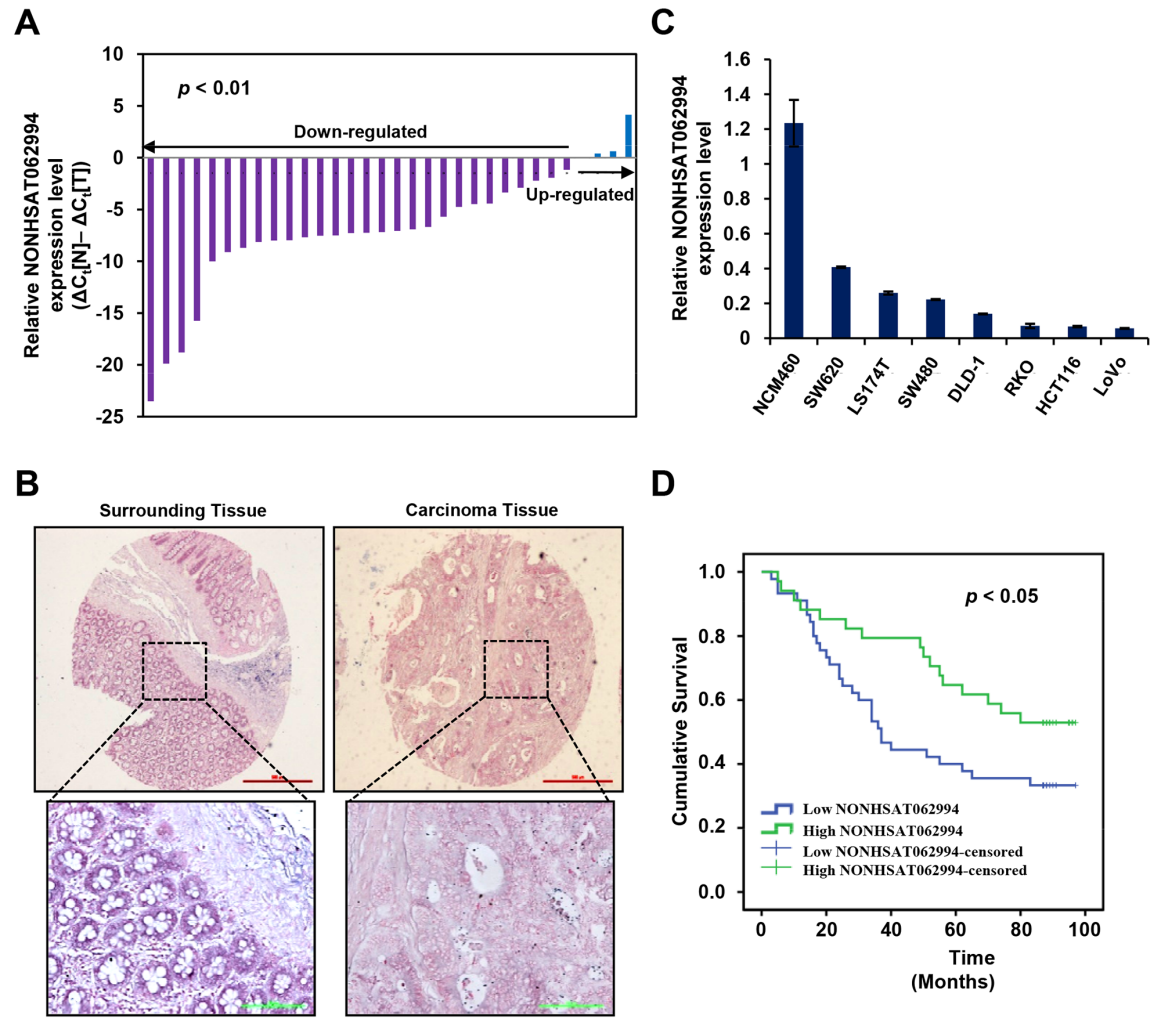
NONHSAT062994 levels are decreased in CRC tissues and are correlated with poor clinical outcome **(A)** NONHSAT062994 expression in 32 CRC samples was evaluated by qPCR. **(B)** ISH for NONHSAT062994 in paraffin-embedded CRC tissues. **(C)** qPCR for *NONHSAT062994* expression in seven CRC cell lines and a normal human colon mucosal epithelial cell line. **(D)** Kaplan-Meier survival curve of CRC patients with low (*n* = 49) and high (*n* = 37) NONHSAT062994 expression.

To define the role of lncRNA NONHSAT062994 in human CRC, NONHSAT062994 expression was measured in 32 pairs of clinical CRC tissues and matched adjacent normal tissue samples using qPCR. NONHSAT062994 transcript levels were significantly lower in CRC tissues compared with paired adjacent normal tissues (Figure 1A). To confirm these results, *in situ* hybridization (ISH) was used to examine NONHSAT062994 expression in 86 CRC tissues; the results revealed that NONHSAT062994 expression was obviously lower in CRC tissues (Figure 1B). Next, NONHSAT062994 expression was measured in 7 human CRC cell lines (SW620, LS174T, SW480, DLD-1, RKO, HCT116, and LoVo) and a normal human colon mucosal epithelial cell line (NCM460). There was less NONHSAT062994 expression in all 7 CRC cell lines compared with NCM460 cells (Figure 1C). These data suggest that lncRNA NONHSAT062994 is expressed at low levels in human CRC tissues and cell lines.

To further understand the significance of NONHSAT062994 expression in CRC, we examined the relationship between NONHSAT062994 expression and the clinicopathological status of patients with CRC. As shown in Table 1, low NONHSAT062994 expression in CRC was significantly correlated with tumor size (*p* = 0.022). However, NONHSAT062994 expression was not associated with other clinicopathological features such as patient gender, age, lymph node metastasis, and TNM stage. In addition, Kaplan-Meier analysis in 86 patients with CRC revealed that low NONHSAT062994 expression in CRC tissues was significantly correlated with reduced OS. The median survival time of CRC patients with low NONHSAT062994 expression was <40 months. However, patients expressing high levels of NONHSAT062994 had a long median survival time (>100 months; Figure 1D). These data suggest that lncRNA NONHSAT062994 plays critical roles in the pathogenesis and prognosis of CRC.

**Table 1:** Correlation of NONHSAT062994 expression with patients’ clinicopathological variables in 86 cases of CRC

Characteristics	All cases(N=86)	NONHSAT062994 Expression (%)			
		Low (n=49)	High (n=37)	χ2 value	*p* value
**Gender**				0.164	0.685
Male	44	26	18		
Female	42	23	19		
**Age (years)**				0.905	0.342
≤ 63	28	18	10		
> 63	58	31	27		
**Tumor size (cm)**				5.217	0.022
< 5	39	17	22		
≥ 5	47	32	15		
**Lymph node metastasis**				0.120	0.729
No	32	19	13		
Yes	54	30	24		
**TNM stage**				0.759	0.384
I/II	56	30	26		
III/IV	30	19	11		

### NONHSAT062994 overexpression inhibits CRC cell proliferation *in vitro* and growth *in vivo*

**Figure 2:**
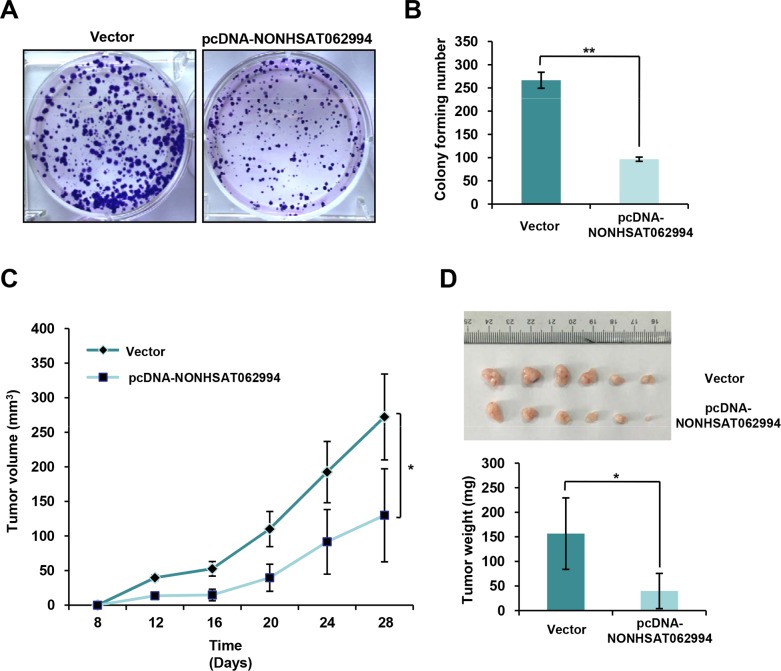
NONHSAT062994 overexpression impairs CRC cell proliferation *in vitro* and growth *in vivo* **(A, B)** Colony formation assays were performed using control or NONHSAT062994-overexpressing HCT116 cells. Representative images are shown (A; magnification, × 1), and the relative colony numbers were counted (B). **(C, D)** NONHSAT062994 overexpression impairs colorectal tumorigenesis. HCT116 cells overexpressing NONHSAT062994 or vector were transplanted into nude mice. The tumor volumes were calculated every 3 days (C). Representative images of tumors and the mean tumor weight in each group are shown (D). Statistical significance was determined using two-tailed unpaired Student's *t* tests. **p < 0.01.

To investigate the potential biological functions of NONHSAT062994 in CRC cells, colony formation assays were performed to assess the effects of NONHSAT062994 overexpression on the proliferation of HCT116 cells. The results showed that the ability of HCT116 cells to form colonies was potently impaired when cells overexpressed NONHSAT062994 (Figure 2A, 2B). Next, the effects of NONHSAT062994 overexpression on CRC growth were assessed in an *in vivo* mouse model. The same number of HCT116 cells overexpressing NONHSAT062994 or control cells were injected into subcutaneous sites of male nude mice. All mice developed xenograft tumors after 7 days. However, NONHSAT062994 overexpression greatly inhibited tumor growth, as revealed by an decreased tumor size (Figure 2C) and weight (Figure 2D) in the overexpression group compared with the control group. These observations suggest that NONHSAT062994 plays a role in colorectal tumorigenesis.

### Knocking down NONHSAT062994 promoted CRC cell proliferation *in vitro* and growth *in vivo*

**Figure 3:**
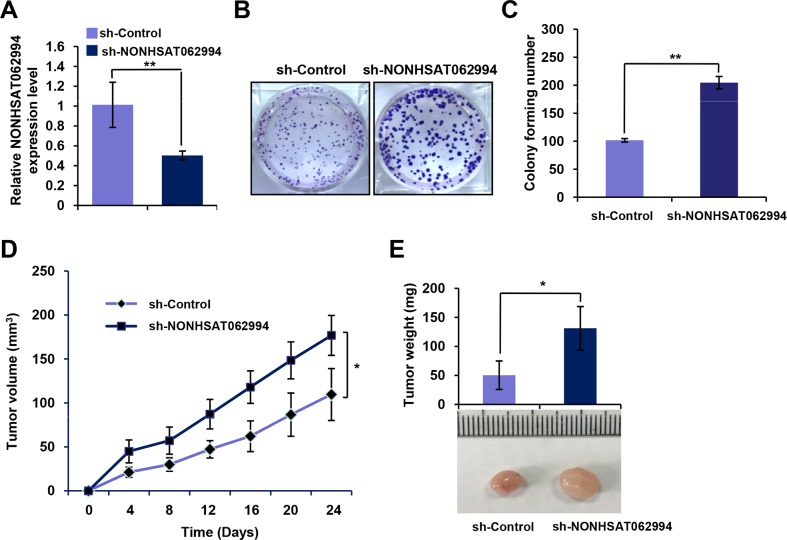
Knocking down NONHSAT062994 promotes CRC growth *in vitro* and *in vivo* **(A)** qPCR for *NONHSAT062994* expression in wild-type CRC cells (shRNA/Control) and CRC cells with stable knockdown of *NONHSAT062994* (shRNA/NONHSAT062994). **(B, C)** Colony formation assays were performed in shRNA/Control or shRNA/NONHSAT062994 CRC cells. Representative images are shown (B; magnification, × 1), and the relative colony numbers were counted (C). **(D, E)** Effects of *NONHSAT062994* knockdown on SW620 cell growth in nude mice. Tumor volumes were measured every 3 days (D). Representative tumor images and the mean tumor weight in each group are shown (E). Statistical significance was determined using two-tailed, unpaired Student's *t* tests. **p < 0.01.

Next, a loss-of-function study was performed to investigate the effects of knocking down NONHSAT062994 on the proliferation and growth of CRC cells. The knockdown efficiency was confirmed using qPCR (Figure 3A). The ability of NONHSAT062994 to affect cell proliferation was assessed using colony formation assays. Knocking down NONHSAT062994 expression significantly enhanced cell proliferation (Figure 3B, 3C) in LS174T cells *in vitro*. In a nude mouse tumor growth model, knocking down NONHSAT062994 expression greatly promoted tumor growth, as evidenced by the increased size (Figure 3D) and weight (Figure 3E) of tumors derived from NONHSAT062994-depleted SW620 cells compared with control cells. Together, these experiments demonstrated that knocking down NONHSAT062994 enhances CRC cell growth i*n vitro* and *in vivo*, strongly suggest that NONHSAT062994 suppressed colorectal tumorigenesis.

### NONHSAT062994 inhibited Akt signaling

**Figure 4:**
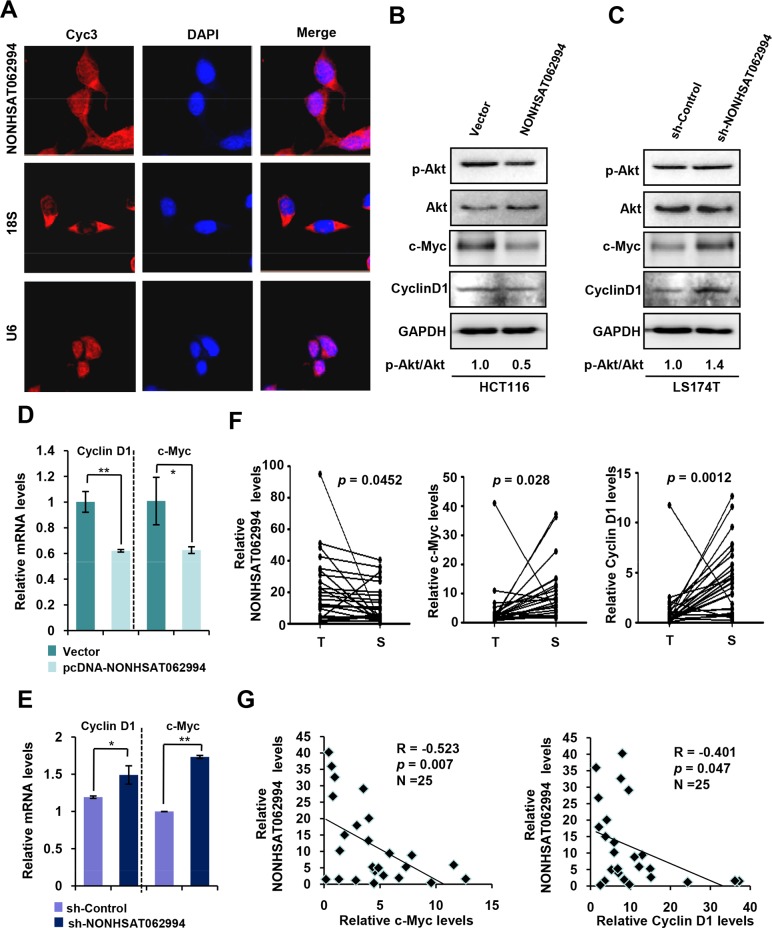
NONHSAT062994 inactivates Akt signaling **(A)** Confocal FISH images showing that NONHSAT062994 (red) was primarily located in the cytoplasm of HCT116 cells. Nuclei were stained with DAPI (blue). Representative images are shown (magnification, ×400). **(B)** NONHSAT062994 overexpression decreased the levels of phosphorylated Akt, c-Myc and Cyclin D1. HCT116 cells were transfected with vector or NONHSAT062994, and total cell lysates analyzed using western blotting to determine phosphorylated Akt, c-Myc and Cyclin D1 levels. **(C)** Silencing NONHSAT062994 increased the levels of phosphorylated Akt, c-Myc and Cyclin D1. LS174T cells were transiently transfected with *NONHSAT062994* siRNA, and total cell lysates were analyzed using western blotting to measure phosphorylated Akt, c-Myc and Cyclin D1 levels. **(D)** NONHSAT062994 overexpression inhibited c-Myc and Cyclin D1 expression. HCT116 cells were transfected with vector or NONHSAT062994, and qPCR was used to determine *c-Myc and Cyclin D1* mRNA levels. Statistical significance was determined using two-tailed unpaired Student's *t* tests. *p < 0.05, **p < 0.01. **(E)** Silencing NONHSAT062994 induces c-Myc and Cyclin D1 expression. SW620 cells were transfected with *NONHSAT062994* shRNA, and qPCR was used to measure *c-Myc* and *Cyclin D1* mRNA levels. Statistical significance was determined using two-tailed, unpaired Student's *t* tests. *p < 0.05, **p < 0.01. **(F)** Comparison of NONHSAT062994, c-Myc, and Cyclin D1 expression in CRC tissues and paired adjacent normal tissues. Statistical significance was determined using two-tailed paired Student's *t* tests. **p < 0.01. **(G)** Spearman's analysis of the correlation between NONHSAT062994 and c-Myc and Cyclin D1 levels in 25 CRC tissues.

Since loss of NONHSAT062994 contributes to CRC proliferation and growth, we next investigated the mechanism by which NONHSAT062994 regulates colorectal tumorigenesis. First, the subcellular localization of NONHSAT062994 was investigated in CRC cells using confocal microscopy with fluorescent *in situ* hybridization (FISH). The results revealed that NONHSAT062994 was primarily located in the cytoplasm (Figure 4A), suggesting that it may exert its biological effects in the cytoplasm.

Akt signaling regulates cell proliferation and growth [7]; thus, we investigated the effects of NONHSAT062994 on Akt signaling. Interestingly, overexpressing NONHSAT062994 in HCT116 cells significantly reduced Akt activation (Figure 4B). Conversely, silencing NONHSAT062994 in LS174T cells that express relatively high levels of NONHSAT062994 greatly enhanced Akt activation (Figure 4C). These results suggest that NONHSAT062994 plays an important role in Akt signaling.

To further validate the regulatory role of NONHSAT062994 on Akt signaling, we next examined whether NONHSAT062994 regulates the expression of c-Myc and Cyclin D1, which are downstream targets of Akt signaling. Western blot and qPCR showed that overexpressing NONHSAT062994 in HCT116 cells markedly reduced c-Myc and Cyclin D1 expression (Figure 4B, 4D). Conversely, silencing NONHSAT062994 in SW620 cells significantly increased c-Myc and Cyclin D1 expression (Figure 4C, 4E). The correlation between NONHSAT062994 and c-Myc and Cyclin D1 expression was further investigated in 25 CRC tissues using qPCR. Lower *NONHSAT062994* expression was observed in CRC tissues compared with paired adjacent normal tissues (Figure 4F). Similarly, the levels of *c-Myc* and *Cyclin D1* were significantly higher in tumors compared with adjacent normal tissues (Figure 4F). Spearman's rank correlation analysis confirmed that there was a significant negative correlation between *NONHSAT062994* and *c-Myc* and *Cyclin D1* expression (Figure 4G). Thus, these observations further confirmed that NONHSAT062994 inhibits Akt signaling in CRC.

### NONHSAT062994 required Akt signaling to suppress colorectal tumorigenesis

**Figure 5:**
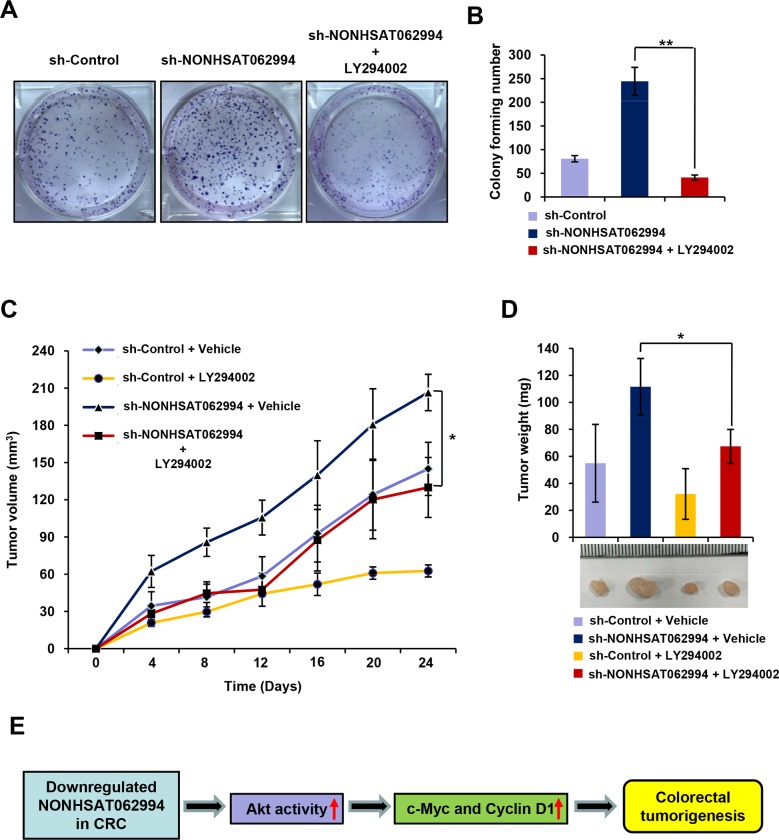
Akt signaling is indispensable for NONHSAT062994-mediated CRC cell proliferation and growth **(A, B)** LY294002 inhibitsNONHSAT062994 silencing-induced CRC cell proliferation. Colony formation assays were performed in NONHSAT062994 shRNA-transduced SW620 cells treated with vehicle or 5 μM LY294002. Representative images are presented (A; magnification, ×1), and the relative number of colonies was counted (B). Statistical significance was determined using two-tailed unpaired Student's *t* tests. **p < 0.01. **(C, D)** LY294002 inhibitsNONHSAT062994 silencing-induced CRC growth. Tumor volumes were measured every 3 days (C). Representative tumor images are shown and the mean tumor weight in each group was measured (D). **(E)** Schematic representation of the function and potential mechanism of NONHSAT062994 in colorectal cancer. Statistical significance was determined using two-tailed unpaired Student's *t* tests. *p < 0.05.

Given the evidence linking aberrant Akt activation to tumorigenesis [7], we next investigated whether Akt signaling is required for the inhibitory effects of NONHSAT062994 on CRC cell proliferation and growth. Colony formation assays revealed that LY294002, an inhibitor of Akt signaling, significantly inhibited NONHSAT062994 silencing-induced CRC cell proliferation (Figure 5A, 5B). The *in vitro* data were then confirmed *in vivo*. Treatment with LY294002 greatly reduced the size (Figure 5C) and weight (Figure 5D) of NONHSAT062994-silenced tumors to a level comparable with the control group. Taken together, these results suggest that the inhibitory effects of NONHSAT062994 on colorectal tumorigenesis are dependent on Akt signaling.

## DISCUSSION

Emerging evidence has implicated aberrant expression of lncRNAs in cancer development and progression [11]. However, it remains unclear whether and how lncRNA NONHSAT062994 regulates cancer development and progression. In the current study, we reported for the first time that downregulation of the lncRNA NONHSAT062994 contributes to colorectal tumorigenesis by inactivating Akt signaling. In addition, NONHSAT062994 was downregulated in human CRC tissues and all CRC cell lines examined, suggesting that NONHSAT062994 may play a critical role in CRC. Analysis of the relationship between NONHSAT062994 expression and the clinical pathological characteristics of 86 CRC patients using tissue microarrays revealed a significant correlation between NONHSAT062994 expression and tumor size, suggesting that NONHS AT062994 plays an important role in colorectal tumorigenesis. Kaplan-Meier analysis revealed that lower NONHSAT062994 levels were closely associated with the OS of CRC patients. Thus, these findings suggest that NONHSAT062994 might be a novel prognostic marker for CRC.

The effects of NONHSAT062994 on CRC cell proliferation and growth was demonstrated directly in our *in vitro* and *in vivo* studies. The results showed that NONHSAT062994 overexpression significantly impaired CRC cell proliferation and markedly inhibited tumor growth. Conversely, silencing NONHSAT062994 markedly promoted CRC cell proliferation and growth both *in vitro* and *in vivo*. Thus, these observations together with the data from the clinical investigation strongly suggest that lncRNA NONHSAT062994 may suppress colorectal tumorigenesis.

Accumulating studies have revealed that Akt signaling plays a crucial role in determining tumorigenesis [18]. Akt signaling is constitutively activated in various cancers including liver cancer, lung cancer, and CRC [19–22], which contributes to cancer development and progression. Several molecules including lncRNAs regulate this signaling. For example, the lncRNA AK023948 promotes Akt activity by functionally interacting with DHX9 and p85 [23]. The lncRNA NEAT1 activates Akt signaling in CRC [24]. The current study found that NONHSAT062994 was primarily located in the cytoplasm of CRC cells, which suggests that NONHSAT062994 could function as an important mediator of signaling transduction in colorectal tumorigenesis. An important observation in the current study is that cytoplasmic NONHSAT062994 functions as a tumor suppressor in CRC by inactivating Akt signaling. *In vitro*, we observed that the ectopic expression of NONHSAT062994 significantly decreased Akt activity. Conversely, silencing NONHSAT062994 increased Akt activity. These data were also validated by clinical observations, which revealed a significant negative correlation between NONHSAT062994 and the expression of c-Myc and Cyclin D1, which are downstream targets of Akt signaling. Inhibiting Akt activity markedly impaired NONHSAT062994 silencing-induced CRC cell proliferation and growth *in vitro* and *in vivo*. Therefore, these results suggest that Akt signaling is required for NONHSAT062994-mediated colorectal tumorigenesis (Figure 5E). However, much more work is required to investigate the detailed mechanisms by which NONHSAT062994 regulates Akt signaling in the future.

In summary, the current study demonstrated that lncRNA NONHSAT062994 is downregulated in CRC tissues and cell lines, and provided the first evidence that NONHSAT062994 inhibits CRC cell proliferation and growth by inactivating Akt signaling. The critical role of NONHSAT062994 in colorectal tumorigenesis provides a candidate prognositc biomarker or a target for CRC prognosis and therapy.

## MATERIALS AND METHODS

### Tissue samples and evaluation

A total of 32 human CRC tissues were obtained from the First Affiliated Hospital of Soochow University (Suzhou, Jiangsu, China) from 2015 to 2016. The expression of NONHSAT062994 was investigated using qPCR and ISH in primary CRC tumors and matched surrounding tissues. The correlation of NONHSAT062994 expression with patients’ clinicopathological variables and patients’ survival outcomes were illustrated using a CRC tissue microarray (Outdo Biotech Co., Ltd, Shanghai, China) containing 86 CRC samples with survival time. These samples were collected between 2006 and 2007. The study was approved by Soochow University for Biomedical Research Ethics Committee, and all of the patients provided informed consent. The clinical characteristics of all patients included in this study are listed in Table 1.

### Antibody and regents

Antibodies against p-Akt (Ser473) (cat. # 4060) and Akt (cat. # 9272) were purchased from Cell Signaling Technology (Beverly, MA, U.S.). Anti-b-actin (cat. # A5441) and LY294002 were purchased from Sigma-Aldrich (St. Louis, MO, U.S.). Lipofectamine 2000 and TRIZOL reagent were purchased from Invitrogen (Carlsbad, CA, USA) and WesternBright ECL reagents were purchased from Advansta (Menlo Park, CA, USA).

### Cell culture

The SW480, HCT116, SW620, LS174T and RKO human CRC cell lines were purchased from the Cell Bank of the Chinese Academy of Sciences (Shanghai, China). These cells were cultured in RPMI-1640 medium containing 10% fetal bovine serum at 37°C in a humidified 5% CO_2_ atmosphere. However, The normal human colon mucosal epithelial cell line NCM460 was maintained in 50% DMEM and 50% RPMI-1640 medium containing 20% fetal bovine serum in a humidified atmosphere containing 5% CO2 at 37°C. All cell lines were passaged as previously described [4, 25].

### Generation of stable cell lines

SW620 cell lines stably expressing NONHSAT 062994-specific shRNA (shRNA/NONHSAT062994) or scrambled shRNA control (shRNA/Control) were constructed using a lentiviral shRNA technique. The human NONHSAT062994 shRNA target sequences were as follows: shRNA/NONHSAT062994, 5’-AGCCTGAG TGACACAATGA-3’`.

### In situ hybridization (ISH) and fluorescent in situ hybridization (FISH)

In situ hybridization (ISH) was performed to detect the expression of NONHSAT062994 using the 5’ and 3’ digoxin-labeled LNATM-modified probe (5’-TG CAGAAATCCACGGGACCAGA-3’) (Exiqon, Vedbaek, Danmark). Briefly, after dewaxing and rehydration, the CRC tissue microarray were digested with proteinase K, fixed in 4% paraformaldehyde, and then hybridized with NONHSAT062994 probe at 55°C overnight. NONHSAT062994 expression was visualized by anti-DIG-AP conjugate antibody and NBT/BCIP substrate (Roche, Basel, Switzerland).

To determine the localization of NONHSAT062994 in CRC cells, we routinely ordered sets of FISH probe mix targeting NONHSAT062994 from commercial sources (RiboBio Co. Ltd., Guangzhou, China). FISH assay was carried out according to the instructions of the manufacturer. The images were taken with a Nikon ECLIPSE Ni scope with color camera and were processed by NIS-Elements D 4.10.00 software.

### Western blot

Western blot was performed as described recently. Briefly, equal amounts of protein (40 μg) was subjected to SDS polyacrylamide gel electrophoresis. The indicated protein expression was detected using primary and secondary antibodies, and visualized using enhanced chemiluminescence reagents and autoradiography. All measurements were performed in triplicate. Representative blots are shown.

### RNA extraction and qPCR analysis

Total RNAs were extracted and reverse transcribed as recently described [3, 26]. qPCR was performed on the Applied Biosystems 7000 Sequence Detection System (Applied Biosystems, Foster City, CA, USA) using power SYBR Green RT-PCR Reagents (Applied Biosystems, Foster City, CA, USA). The pecific primers for NONHSAT062994 as follows: forward, 5’-TCT GGTCCCGTGGATTTCTG-3’; reverse, 5’-ATCGCCAT CACTGTCCTTCTG-3’.

### Colony formation assay

CRC cells were trypsinized into single-cell suspensions, then 0.5 × 10^3^ cells were placed into each well of a 6-well plate and were cultured in RPMI-1640 medium containing 10% fetal bovine serum for 10-14 days, and then fixed and stained with Wright-Giemsa. The number of foci containing > 50 cells was counted.

### Animal experiments

Nude mice (4–5 weeks old, male, BALB/c) were injected subcutaneously with 2 × 10^6^ CRC cells (n = 6 mice per group). After 7 days of transplantation, the size of the tumor was measured every 3 days. Tumors were removed for assessments after 4 weeks. For LY294002 treatment, LY294002 (25 mg/kg) was intraperitoneally injected into the nude mice every other day. The tumor volume was measured every four days. All animal experiments were approved by the Animal Care and Use Committee of Soochow University.

### Statistical analysis

Each assay was performed in three independent experiments. Data were presented as mean ± s.d. Statistical significance was analyzed using Student's *t*-test (unpaired, two-tailed). Pearson's chi-square test was used to analyse the relationships between NONHSAT062994 expression and CRC patients’ clinicopathological factors, and Spearman's rank Correlation analysis was used to calculate the correlations between the expression levels of NONHSAT062994 and c-Myc and Cyclin D1. The Kaplan-Meier survival analysis was used to illustrate the prognostic relevance of NONHSAT062994 in univariate analysis. *p* < 0.05 was considered statistically significant.

## Abbreviations

lncRNA: long noncoding RNAs (lncRNAs)
qPCR: Quantitative PCR
CRC: Colorectal Cancer
ISH: In Situ Hybridization
FISH: fluorescent in situ hybridization
